# Cost-effectiveness of introducing a maternal vaccine or long-acting monoclonal antibody to prevent infant respiratory syncytial virus disease in Nepal

**DOI:** 10.7189/jogh.15.04292

**Published:** 2025-11-14

**Authors:** Neele Rave, Arun K Sharma, Ram H Chapagain, An Nguyen, Clint Pecenka, Farina L Shaaban, Louis J Bont, Andrew Clark, RSV GOLD III – Health Economics Study Group, Louis Bont, Neele Rave, Farina Leonie Shaaban, Frédéric Debellut, Norbert Fuhngwa, Henshaw Mandi, Rosemary Akuaku, Joycelyn Dame, Amma Ekem, Bamenla Goka, Ebenezer Ntow, Kwabena A Osman, Elias Manjate, Yara Manjate, Izilda Matimbe, Tufária Mussá, Cesar Palha, Cristina Sinussene, Farida Zavala, Esperança Lourenço Guimarães, Assucênio Chissaque, Braiton Maculuve, Tufária Mussá, Cesar Palha, Nilsa de Deus, Mirela Pale, Fadlulai Abdu-Raheem, Anas Abubakar, Abdullahi Aminu, Maria Ahuoiza Garba, Fatima Jummai Giwa, Habiba Lawal, Aira Abiola Olorukooba, Maria Ahuoiza Garba, Fatima Giwa, Bernsah Damian Lawong, Abdullahi Musa, Teddy Naddumba, Aira Abiola Olorukooba, Ram H Chapagain, Rita Dhital, Prakash Joshi, Ranju Karki, Adita Nepali, Arun K Sharma, Rupesh Shrestha, Nirasta Thakili, Ram H Chapagain, Upendra Dhungana, Prakash Joshi, Uttam Paudel, Arun K Sharma, Rupesh Shrestha, Andrew Clark, An Nguyen, Clint Pecenka

**Affiliations:** 1 Department of Paediatrics, University Medical Centre Utrecht, Utrecht, the Netherlands; 2 Department of Paediatrics, Institute of Medicine, Tribhuvan University Teaching Hospital, Kathmandu, Nepal; 3 Department of Paediatrics, Kanti Children's Hospital, Kathmandu, Nepal; 4 PATH, Center for Vaccine Innovation and Access, Ho Chi Minh City, Vietnam; 5 PATH, Center for Vaccine Innovation and Access, Seattle, Washington, USA; 6 Nepal Paediatric Society, Kathmandu, Nepal; 7 Department of Health Services Research and Policy, London School of Hygiene & Tropical Medicine, London, UK

## Abstract

**Background:**

The World Health Organization recommends two passive immunisation strategies to prevent respiratory syncytial virus (RSV) disease in young infants. Both are being introduced in high-income settings, but their affordability and cost-effectiveness have not been evaluated in many low- and middle-income countries. Preliminary estimates of cost-effectiveness are needed to guide immunisation policy and planning in Nepal.

**Methods:**

We estimated the potential health impact and cost-effectiveness of introducing a maternal vaccine (RSVpreF) or long-acting infant monoclonal antibody (mAb) (nirsevimab) over the period 2025–34 in Nepal. We compared both interventions to the *status quo* (no intervention) and to each other. Model inputs included health care cost estimates from a recent prospective cost-of-illness study in Kathmandu, as well as the latest efficacy data from clinical trials. The primary outcome measure was the incremental cost (2023 USD) per disability-adjusted life year (DALY) averted from a governmental health perspective. We conducted a range of deterministic analyses, including scenarios that incorporated a societal perspective and a seasonal approach. Additionally, we performed probabilistic uncertainty analyses to assess decision uncertainty and estimated the likelihood of cost-effectiveness for each intervention across a range of willingness-to-pay thresholds.

**Results:**

Introducing a maternal vaccine (USD 5/dose, 81% coverage, 69% efficacy, 6 months protection) or long-acting infant mAb (USD 5/dose, 97% coverage, 77% efficacy, 5 months protection) could prevent >2300 deaths and >50 000 hospital admissions over ten years. The discounted immunisation programme costs were estimated to be USD 30 and USD 35 million, respectively. Compared to the *status quo*, the maternal vaccine and the long-acting infant mAb were estimated to cost USD 387 and USD 486 per DALY averted, respectively, which is around 0.3 times and 0.4 times the national gross domestic product (GDP) per capita. There was a 95% probability that the maternal vaccine would be cost-effective at USD 5 per dose, assuming a willingness-to-pay threshold of 0.5 times the national GDP per capita. With our base case assumptions, the maternal vaccine dominated the mAb (*i.e.* generated more health benefits at a lower cost). However, the results (and the rank order of interventions) were sensitive to the dose price, efficacy, duration of protection, and RSV disease burden estimates. Cost-effectiveness of the mAb improves with timely administration or when a seasonal approach is implemented.

**Conclusions:**

New passive immunisation strategies have the potential to prevent a substantial number of RSV-related hospitalisations and deaths in Nepal. Cost-effectiveness and product choice will heavily depend on the price negotiated for each product.

Acute lower respiratory infections (ALRIs) caused by respiratory syncytial virus (RSV) are a major cause of death and hospital admissions among young children. In 2019, RSV was estimated to cause 33 million cases and 118 000 deaths in children under five years, with more than 95% of the burden in low- and middle-income countries (LMICs) [1]. In 2017, the global economic burden of managing RSV infections in young children was estimated to be USD 5.5 billion (EUR 4.82 billion (95% confidence interval (CI) = EUR 3.47–7.93 billion)), with 65% of the economic burden in LMICs [2]. Despite this, most cost-effectiveness studies to date have been conducted in high-income countries, limiting their relevance for health policy decision-making in LMICs.

Two passive RSV immunisation strategies have recently received market approval and are recommended for global use by the World Health Organization (WHO) [3]. A maternal vaccine (RSVpreF, Abrysvo, Pfizer) has demonstrated efficacy of 69.4% (97.58% CI = 44.3–84.1) against severe medically attended RSV-ALRI after 180 days of follow-up [4]. In addition, a long-acting infant monoclonal antibody (mAb) (nirsevimab, Beyfortus, AstraZeneca and Sanofi) [5] has demonstrated efficacy of 76.8% (95% CI = 49.4–89.4) against hospitalisation for RSV-associated lower respiratory tract infection after 150 days of follow-up [6]. Both intervention strategies have already been introduced in national immunisation programmes in parts of the Americas and Europe and are starting to be considered for introduction in LMICs.

Health systems in LMICs vary widely in terms of service delivery, health care financing, disease burden, and available infrastructure. These differences influence how interventions are implemented, how costs are incurred, and how health outcomes are realised. As a result, cost-effectiveness findings from high-income countries, and even from other LMICs, may not be directly applicable, underscoring the need for country-specific analyses to support evidence-based policy decisions.

In Nepal, RSV is one of the leading respiratory viral pathogens among young children presenting with respiratory symptoms, both in rural and urban areas [7–9]. In a semi-urban region, 15% of all pneumonia cases in children under three years of age were associated with RSV. In urban areas, 28% of ALRI patients in intensive care units (ICUs) tested positive for RSV [10], and a recent study detected RSV in 73% of hospitalised children with severe ALRI during the respiratory season [9].

Nepal’s National Immunisation Program has recently integrated several new vaccines into the national immunisation schedule, including the rotavirus and typhoid conjugate vaccine [11]. The country’s vaccination initiatives are strongly supported by Gavi, the Vaccine Alliance, and the United Nations Children’s Fund (UNICEF), which provide both financial and technical assistance. In February 2025, Nepal launched a nationwide human papillomavirus vaccination campaign with support from Gavi [12]. Since Gavi’s Vaccine Investment Strategy has prioritised RSV vaccines for the 2021–25 funding period and beyond, RSV is considered a potential candidate for future inclusion in Nepal’s National Immunisation Program. In March 2025, the first maternal RSV vaccine received WHO prequalification, and shortly thereafter, Gavi approved funding for country introductions [13,14]. These developments highlight the need for national decision-makers in Nepal to have timely estimates of the potential health impact and cost-effectiveness of both the maternal vaccine and long-acting infant mAb.

We aim to evaluate the potential health and economic impact of a maternal vaccine and a long-acting infant mAb, utilising the best available evidence for the Nepali context. The findings are expected to provide essential information for informed national policy decisions on implementing these interventions.

## METHODS

### Study design

We estimated the potential health impact and cost-effectiveness of two passive RSV immunisation strategies for children under five years of age in Nepal over 10 years (2025–34). We compared a maternal vaccine (RSVpreF, Abrysvo, Pfizer) and long-acting infant mAb (nirsevimab, Beyfortus, AstraZeneca and Sanofi) to the *status quo* (no pharmaceutical intervention) and to each other. The primary outcome measure was the cost (2023 USD) per disability-adjusted life year (DALY) averted, from a governmental health perspective. We applied a 3% discount rate to both the costs and benefits of immunisation following WHO guidelines [15]. To interpret the cost-effectiveness ratios (USD per DALY averted) we used a willingness-to-pay (WTP) threshold to represent what the government might be willing to pay for each DALY averted by the intervention. In the absence of a country-specific WTP threshold for Nepal, we used 0.34 times the national gross domestic product (GDP) per capita. This value represents the midpoint of the 0.28–0.39 range estimated by Ochalek *et al.*, who estimated opportunity costs specific to Nepal by using data on current health expenditures and health outcomes [16]. This approach seeks to quantify the marginal productivity of health spending, to account for the health that is forgone elsewhere in the system when resources are reallocated. This threshold is consistent with the estimates from Pichon-Riviere *et al.*, who used an independent, but similar, methodology and estimated a threshold of 0.35 (95% CI = 0.18–0.44) for Nepal [17]. To reflect broader global health decision-making practices and account for uncertainty in the threshold, we also evaluated the probability that the RSV intervention strategies would be cost-effective at two alternative thresholds of 0.5 and 1 times the GDP per capita [18]. We assumed a GDP per capita of USD 1324 for Nepal, based on estimates from the World Bank for the year 2023 [19]. We reported all costs in 2023 USD (USD 1 = NPR 132.12) [20].

### Model

To calculate the potential health impact and cost-effectiveness of both RSV intervention strategies, we used the universal vaccine decision-support model (UNIVAC, version 1.7) a static proportionate outcomes cohort model. The model can be used to provide transparent and conservative estimates of the projected impact and cost-effectiveness of the maternal vaccine and long-acting infant mAb [21]. It was recently applied in Argentina, Mozambique, and Vietnam [22–24]. The model is populated with parameters on population demographics, rates of RSV disease (cases, clinic visits, hospital admissions, and deaths before the age of five), RSV health care costs, intervention efficacy, coverage, timeliness, and programme costs.

To build consensus on the inputs used in the UNIVAC model, we consulted key national stakeholders, including representatives from Nepal’s Ministry of Health and Population (Family Welfare, Epidemiology and Disease Control, Logistics, Procurement and Management, Finance, and Policy and Planning Divisions), the National Immunization Technical Advisory Group, and international partners (WHO and UNICEF) (**Table 1**). Professional bodies, including the Nepal Paediatric Society and paediatric nursing associations, also contributed. Stakeholder engagement was conducted between September 2023 and August 2024 through a combination of both in-person meetings and structured discussions. These interactions provided critical insights into available country-specific data and guided decisions on treatment-seeking behaviour, health care utilisation patterns, and the operational feasibility of vaccine delivery. We iteratively incorporated stakeholder feedback to ensure that the model inputs accurately reflect the local context.

**Table 1:** Input parameters for estimating the impact and cost-effectiveness of a maternal vaccine and a long-acting infant mAb for RSV prevention in Nepal

	Central value	UR	Sources
**Incidence rate per 100 000 under-five children per year**			
Non-severe RSV cases	7313	3269–9140	Chu *et al.* [7], Li *et al.* [1]
Non-severe RSV visits	1901	850–2377	DHS 2022 [25], MICS 2019 [26]
Severe RSV cases	963	431–1204	Chu *et al.* [7], Li *et al.* [1]
Severe RSV visits	2167	969–2708	DHS 2022 [25], MICS 2019 [26]
Severe RSV hospitalisation	722	323–903	DHS 2022 [25]
RSV deaths	44	19–54	Li *et al.* [1], GOLD-ICU [10]
**Non-severe cumulative age-specific burden, %**			
<1 month	1	NA	GOLD-COI [9]
<2 months	3		
<3 months	8		
<6 months	21		
<1 year	47		
<2 years	77		
<3 years	90		
<4 years	96		
<5 years	100		
**Severe cumulative age-specific burden, %**			
<1 month	7	NA	GOLD-COI [9]
<2 months	8		
<3 months	24		
<6 months	44		
<1 year	69		
<2 years	89		
<3 years	96		
<4 years	99		
<5 years	100		
**Cumulative age-specific mortality burden, %**			
<1 month	16	NA	GOLD-COI [9]
<2 months	15		
<3 months	44		
<6 months	64		
<1 year	80		
<2 years	91		
<3 years	96		
<4 years	98		
<5 years	100		
**Disability weights, %**			
Non-severe RSV	5.1	3.2–7.4	GBD 2019 [27]
Severe	13.3	8.8–19.0	GBD 2019 [27]
**Mean duration of illness, in days**			
Non-severe RSV	5	3–7	Graham and Anderson [28]
Severe RSV	10	7–14	Graham and Anderson [28]
**Maternal immunisation impact**			
Expected coverage (ANC proxy), %	81.0	77.8–92.0	DHS 2022 [25]
Efficacy against RSV severe cases, %	69.4	44.3–84.1	Kampmann *et al.* [4]
Efficacy against RSV non-severe cases, %	51.3	29.4–66.8	Kampmann *et al.* [4]
Duration of protection (months)	6	5–7	Kampmann *et al.* [4]
**Infant mAb impact, in USD**			
Expected coverage (BCG proxy), %*	97	95–99	WHO and UNICEF [29]
Efficacy against RSV severe cases, %	76.8	49.4–89.4	Muller *et al.* [6]
Efficacy against non-severe RSV, %	76.4	62.3–85.2	Muller *et al.* [6]
Duration of protection (months)	5	4–6	Muller *et al.* [6]
**Costs of intervention, in USD**			
Price per dose†	5	NA	Assumption
Syringe price per dose	0.000	NA	Assume pre-filled syringe
Safety box price per dose	0.005	NA	UNICEF [30]
International handling (% price per dose)	3	3–5	UNICEF [31]
International delivery (% price per dose)	6	2–15	Debellut [32]
Incremental delivery cost per dose†	1.66	1.00–2.75	Janoch *et al.* [33]
**Wastage assumptions, % per dose**			
Wastage of vaccines and syringes	5	NA	Assumption
Safety boxes/bags	5	NA	Assumption
**Costs of RSV-related care governmental perspective, in USD†**			
Non-severe disease			
*Outpatient visit cost*	15.22	14.51–15.94	GOLD-COI [9], MoHP [34]
Severe disease			
*Outpatient visit cost*	15.22	14.51–15.94	GOLD-COI [9], MoHP [34]
*Hospitalisation cost*	47.65	44.21–66.38	GOLD-COI [9], MoHP [34]
**Costs of RSV-related care societal perspective, in USD†**			
Non-severe disease			
*Outpatient visit cost*	41.90	23.90–59.91	GOLD-COI [9], MoHP [34]
Severe disease			
*Outpatient visit cost*	41.90	23.90–59.91	GOLD-COI [9], MoHP [34]
*Hospitalisation cost*	273.61	251.22–295.99	GOLD-COI [9], MoHP [34]

ANC – antenatal care, BCG – Bacillus Calmette-Guerin, COI – cost-of-illness, DHS – Demographic and Health Surveys, mAb – monoclonal antibody, ICU – intensive care unit, MICS – Multiple Indicator Cluster Surveys, MoHP – Ministry of Health and Population, RSV – respiratory syncytial virus, UNICEF – United Nations Children’s Fund, UR – uncertainty range, WHO – World Health Organization

*BCG vaccination real-world timeliness is applied to the coverage assumption for the long-acting infant mAb. For simplicity, no age restriction was assumed for the long-acting infant mAb administration.

†All future costs and benefits are discounted at a 3% discount rate per year. Costs and gross domestic product per capita are expressed in 2023 USD.

### Burden of RSV disease

We estimated the rate of RSV disease events (cases, visits, clinic visits, hospital admissions, deaths) per 100 000 per year (<5 years) and applied these to United Nations World Population Prospects estimates of life-years lived between birth and five years in each evaluated birth cohort. We assumed these rates would remain fixed in the absence of new pharmaceutical interventions (except RSV deaths), which we conservatively assumed would decrease at the same rate as the United Nations World Population Prospects projected improvements in overall under-five mortality.

We assumed annual incidence of 7313 per 100 000 < 5 years for non-severe RSV cases, and 963 for severe RSV cases based on a cohort study by Chu *et al*. in the rural sub-tropical plains of Nepal [7]. This study captured incidence in children under six months of age, so we adjusted the incidence rate to reflect children under five by assuming that 22% of under-five cases would be aged under six months, based on preliminary estimates from a systematic review evaluating RSV age distribution in LMICs (data not published). We assumed that 12% of RSV cases would be severe, consistent with estimates from Chu *et al.* [7] and Li *et al.* [1]. We assumed 0.26 clinic visits per non-severe RSV case based on the proportion of acute respiratory infection cases with access to public sector providers, as reported in the 2019 Nepal Multiple Indicator Cluster Surveys (MICS 2019) and the 2022 Demographic and Health Survey (DHS 2022) [25,26]. For severe RSV cases, we assumed that 75% would have access to a hospital, based on the reported access to care for acute respiratory infections in the DHS 2022 [25]. We further assumed three clinic visits per hospital admission because caregivers in Nepal often return to health care facilities multiple times when symptoms persist or worsen, and follow-up care is typically provided after hospital discharge. We assumed a hospital fatality ratio of 2%, reflecting the average of two hospital fatality ratio estimates from LMICs [1,8] and that 67.5% of RSV deaths would occur in the community, reflecting the average of two separate estimates from LMICs [1,24]. Our estimates of the annual number of RSV deaths were higher than estimates derived using a separate ‛top-down’ (proportionate mortality) approach (1285 *vs.* 643). We used the higher burden estimate derived from the ‛bottom-up’ (incidence-based) approach in the base case scenario to avoid underestimating the potential impact of RSV, assuming that registration systems are often incomplete and community deaths may be underreported. To account for this uncertainty, we ran a scenario analysis using both the lower and higher mortality estimates, including an even more conservative lower bound of 561 based on Li *et al*. We incorporated these mortality bounds into the lower and higher burden scenarios, respectively, and formed the range used in the probabilistic uncertainty analysis (Appendix S1 in the **Online Supplementary Document**).

We obtained the RSV age-burden distribution from a recent cost-of-illness (COI) study [9] and an associated study on high-dependency units and ICUs in Kathmandu, Nepal [10] (Appendix S2 in the **Online Supplementary Document**). The COI study included all children under two years with ALRI who visited Kanti Children’s Hospital or Tribhuvan Teaching Hospital between July and November 2023. The ICU study focused on life-threatening cases being admitted to the high-dependency units/ICUs during two local RSV seasons. We estimated the proportion of under-five RSV counts that were under two years of age based on estimates from a systematic review of RSV age distributions in LMICs (data not published). We used the age distribution of RSV outpatients from the COI study to estimate the age distribution of non-severe RSV cases and clinic visits. We used the age distribution of RSV inpatients from the same study to estimate the age distribution of severe RSV cases, clinic visits, and hospital admissions, and the age distribution of RSV ICU cases from the ICU study as a proxy for the age distribution of RSV deaths. The age distribution for RSV non-severe disease was consistent with that obtained for community cases in more rural areas of southern Nepal [7] and Bhaktapur [8].

### Economic burden of RSV disease

In the base-case scenario, we used cost data from a recent COI study on RSV conducted in Kathmandu from July 2023 to November 2023 [9] (Appendix S3 in the **Online Supplementary Document**). Governmental costs included all expenses incurred by the government in providing services and infrastructure for children with an RSV infection during the full illness episode, as well as direct medical costs borne by the government. We conducted separate scenario analyses from the societal health perspective for completeness. From a societal health perspective, the costs included all government health costs as well as direct medical expenses (*e.g.* medication, imaging), direct non-medical expenses (*e.g.* transportation, food), and indirect costs (*e.g.* lost income) that were borne by households during the initial visit at a governmental health facility and prior/post visit at any health care facility (public or private).

### RSV intervention impact

We calculated the health impact of each intervention by multiplying the expected number of RSV disease events in each week of age under five years by the corresponding coverage and efficacy in the same week of age. In the base-case scenario, we assumed year-round immunisation and maternal vaccine coverage of 81.0% based on antenatal care attendance reported by women who attended four or more antenatal care visits, as documented in DHS 2022 [25]. For the uncertainty range, we assumed a lower coverage of 77.8%, based on the MICS 2019 [26], and an upper coverage of 92%, based on the second dose of diphtheria-tetanus-pertussis vaccine [28].

To estimate the coverage of year-round administration of a long-acting infant mAb, we used the coverage of the Bacillus Calmette-Guérin (BCG) vaccine as a proxy (97%), based on WHO and UNICEF estimates of immunisation coverage for the year 2023 [28]. We assumed an uncertainty range of 2% above and below the point estimate. Birth doses that are not administered on time could result in a reduced impact of the infant mAb; therefore, we assumed that the long-acting infant mAb would be administered with the same delays (coverage by week of age) as reported for BCG in DHS 2022.

We derived efficacy estimates for each RSV intervention from evidence obtained in phase III clinical trials. For the maternal vaccine, an efficacy of 69.4% (97.58% CI = 44.3–84.1) was observed in preventing severe lower respiratory tract infection associated with RSV during the first six months of life [4]. For the long-acting infant mAb, efficacy against very severe disease was assumed to be 76.8% (95% CI: 49.4–89.4) five months after immunisation [6]. For non-severe cases, we assumed the efficacy to be 51.3% (97.58% CI = 29.4–66.8) for the maternal vaccine [4] and 76.4% (95% CI = 62.3–85.2) for the long-acting infant mAb based on any medically attended RSV [6]. Mahmud *et al.* [21] described the efficacy and waning assumptions we applied in our analysis in detail.

### RSV intervention programme costs

The price of both interventions is highly uncertain in LMICs. Therefore, we evaluated three different price scenarios, with USD 5 per dose for the base-case scenario, as well as USD 15 and USD 25 per dose. We assumed these prices would stay constant throughout the ten years. Both the long-acting infant mAb and maternal vaccine are available in a pre-filled syringe [35,36], so we assumed no additional syringe costs. Programme costs included all expenses related to procuring and administering either the maternal vaccine or the long-acting infant mAb. We assumed a 5% wastage for doses and safety boxes.

### Uncertainty analysis

To assess the influence of different parameters and assumptions on the cost-effectiveness results, we ran the following deterministic scenarios:

1. Lower and higher bound rates of annual RSV burden;
2. Lower and higher bound rates of immunisation efficacy (95% CI);
3. High initial immunisation efficacy that gradually decreases to zero by age 12 months [21];
4. Lower and higher bound rates of the incremental health system cost per dose (25th and 75th percentile);
5. Lower and higher bound rates of the RSV cost per visit/admission;
6. Dose prices of USD 15 and USD 25;
7. A societal perspective, taking access to private health facilities into account (overall access: 82%) [26] and including all scaled societal costs from the COI study [9];
8. A societal perspective, taking access to private health facilities and societal costs into account, while excluding indirect costs (*i.e.* lost wages), which accounted for 31% of outpatient costs and 39% of inpatient costs in the COI study [9]; and
9. A seasonal dose administration for both interventions (Appendix S4 in the **Online Supplementary Document**).

We used burden estimates from Li *et al.* [1] as the lower bound and a value of 25% above the adjusted estimate from Chu *et al.* [7] as the upper bound, to reflect uncertainty in incidence rates (**Table 1**). We also conducted a probabilistic sensitivity analysis by simultaneously varying all parameters within their respective ranges through 1000 Monte Carlo simulations. For both interventions, we performed a separate probabilistic sensitivity analysis for three fixed price scenarios (USD 5, USD 15, and USD 25 per dose). For simplicity, we assumed a consistent Beta-PERT distribution for all parameters and their respective ranges. We displayed the probabilistic results on a cost-effectiveness plane. We generated cost-effectiveness acceptability curves to calculate the probability that each intervention would be cost-effective at various WTP thresholds.

## RESULTS

### Lifetime costs and effects based on central input parameters

Over ten years, either the maternal vaccine (USD 5/dose, 81% coverage, 69% efficacy, 6 months protection) or a long-acting infant mAb (USD 5/dose, 97% coverage, 77% efficacy, 5 months protection) could prevent over 2300 deaths and 50 000 hospital admissions, compared to the* status quo* (**Table 2**). The long-acting infant mAb would prevent more visits and hospitalisations compared to the maternal vaccine. Accounting for real-world delays in mAb administration and the fact that RSV mortality peaks early in infancy, the maternal vaccine was estimated to prevent a greater number of RSV deaths (n = 2507; 35.8%) than the long-acting infant mAb (n = 2369; 33.8%), despite the lower coverage assumed for the maternal vaccine. The maternal vaccine, therefore, dominated the mAb (generated more health benefits at lower cost) under our base case assumptions.

**Table 2:** Estimated health impact and costs of RSV preventive interventions compared to no pharmaceutical intervention in Nepal (2025–34)*

	No intervention	Maternal vaccine	mAb
**Lifetime costs and effects**			
Non-severe RSV cases	2 040 658	1 860 216	1 741 341
Non-severe RSV clinic visits	530 465	483 560	452 658
Severe RSV cases	268 721	201 983	196 843
Severe RSV clinic visits	604 691	454 513	442 948
Severe RSV hospital admissions	201 471	151 435	147 581
Severe RSV deaths	7002	4494	4633
DALYs†	181 489	116 210	119 705
Vaccine programme costs†	0	29 938 203	35 397 868
Governmental healthcare costs†	22 610 244	17 935 236	17 215 204
Societal healthcare costs†	85 589 768	67 531 229	65 073 041
**Differences**‡			
Non-severe RSV cases		180 441	299 317
Non-severe RSV clinic visits		46 905	77 807
Severe RSV cases		66 738	71 878
Severe RSV clinic visits		150 178	161 743
Severe RSV hospital admissions		50 036	53 889
Severe RSV deaths		2507	2369
DALYs†		62 279	61 784
Vaccine programme costs†		29 938 203	35 397 868
Government healthcare costs†		4 675 008	5 395 040
Societal healthcare costs†		19 058 538	21 516 726
**Cost per DALY averted†‡**			
Government cost perspective			
*Cost, in USD*		29 938 203	35 397 868
*DALYs averted*		65 279	61 784
*Cost per DALY averted, in USD*		387	486
Societal cost perspective			
*Cost, in USD*		29 938 203	35 397 868
*DALYs averted*		65 279	61 784
*Cost per DALY averted, in USD*		167	225

DALY – disability-adjusted life year, GDP – gross domestic product, mAb – monoclonal antibody, RSV – respiratory syncytial virus

*Maternal vaccine is USD 5 per dose, 81% coverage, 69.4% efficacy, and six months protection, while infant mAb is USD 5 per dose, 97% coverage, 76.8% efficacy, and five months protection.

†All future costs and benefits are discounted at a 3% discount rate per year. Costs and gross domestic product per capita are expressed in 2023 USD.

‡No pharmaceutical intervention was used as a comparator.

Introducing each intervention would have discounted programme costs of USD 30 and USD 35 million in ten years, respectively. Approximately 15% of the programme costs were estimated to be offset by government health care cost savings. From a societal health perspective, 60% of the programme costs would be offset by health care cost savings. Compared to the *status quo*, the maternal vaccine would cost USD 387 per DALY averted, corresponding to 0.29 of the national GDP per capita, and the long-acting infant mAb would cost USD 486 per DALY averted, corresponding to 0.37 of the national GDP per capita.

### Scenario and sensitivity analysis

**Figure 1:**
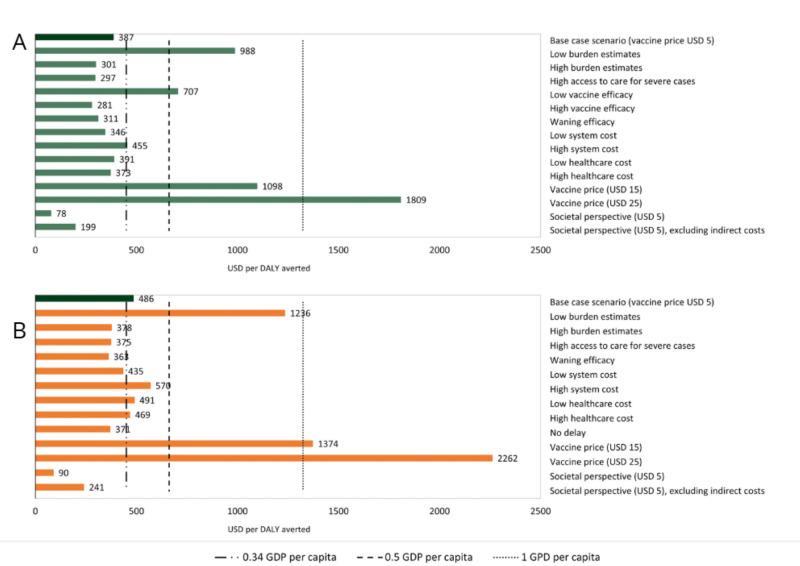
USD per DALY averted for several alternative scenarios. **Panel A.** Maternal vaccine. **Panel B.** Long-acting infant mAb. DALY – disability-adjusted life year, GDP – gross domestic product.

In the base-case scenario, the long-acting infant mAb had a less favourable cost-effectiveness ratio compared to* status quo *than the maternal vaccine (USD 486 *vs.* USD 387 DALYs averted) (**Figure 1**, Panels A and B). Only the maternal vaccine was cost-effective at a WTP threshold of 0.34 times GDP per capita, while the long-acting infant mAb was cost-effective at 0.5 times the GDP per capita in Nepal. However, when adjusting for timely immunisation coverage (no delay), the long-acting infant mAb had more favourable cost-effectiveness compared to *status quo* than the maternal vaccine (USD 371 *vs.* USD 387). When assuming high initial efficacy that waned to zero by 12 months of age, the cost-effectiveness for both interventions decreased to 0.23 times the GDP per capita and 0.27 times the GDP per capita, respectively. Stepwise increases in the price per dose (USD 15 and USD 25) reduced cost-effectiveness, and neither of these dose prices was cost-effective at 0.34 times the GDP per capita.

### Cost-effectiveness at different WTP thresholds

**Figure 2:**
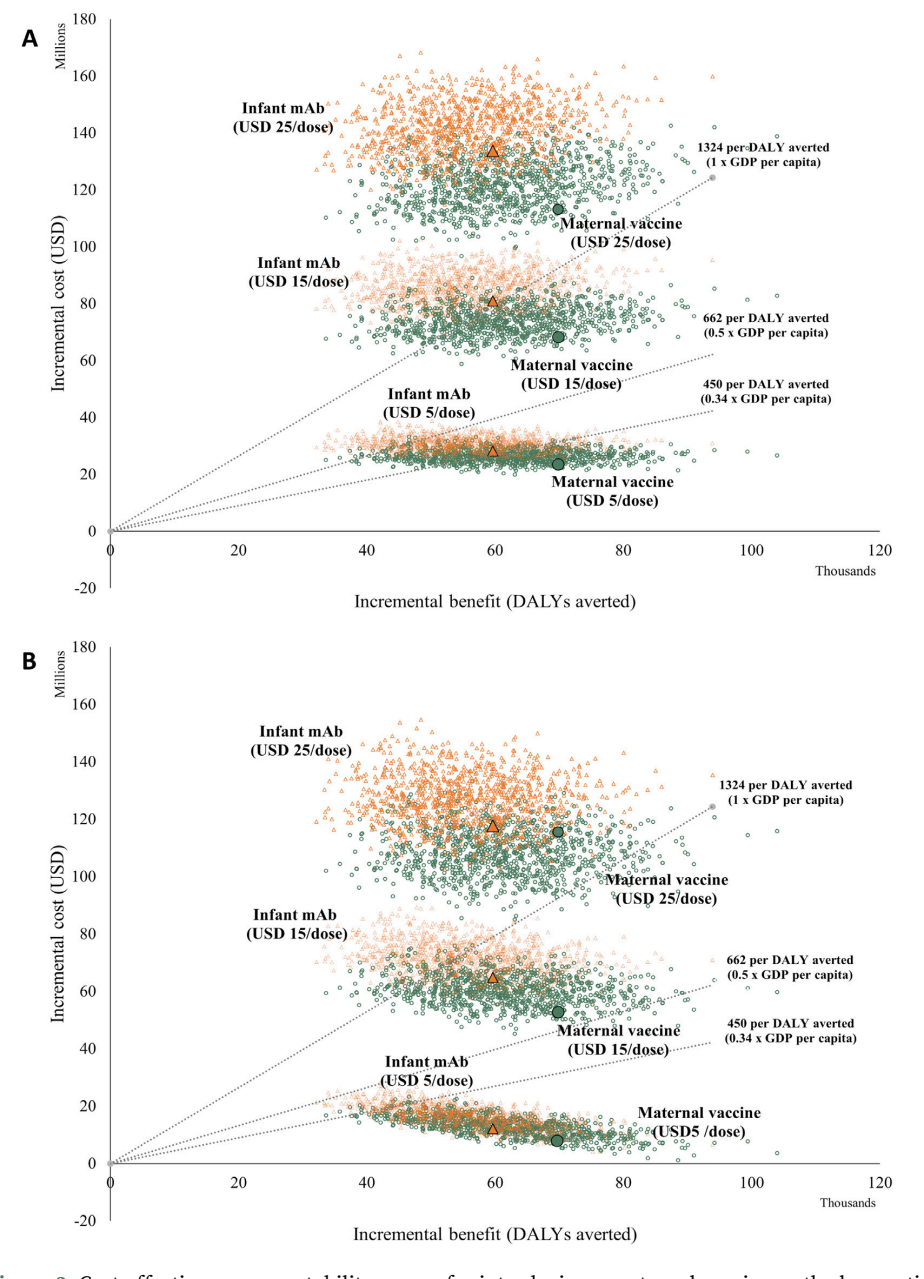
Probabilistic clouds of incremental costs and benefits of both RSV intervention strategies, assuming three different dose prices. **Panel A.** Governmental health perspective. **Panel B.** Societal health perspective. DALY – disability-adjusted life year, mAb – monoclonal antibody, RSV – respiratory syncytial virus.

At all dose prices, the maternal vaccine demonstrated greater incremental health benefits compared to the long-acting infant mAb (**Figure 2**, Panels A and B).

**Figure 3:**
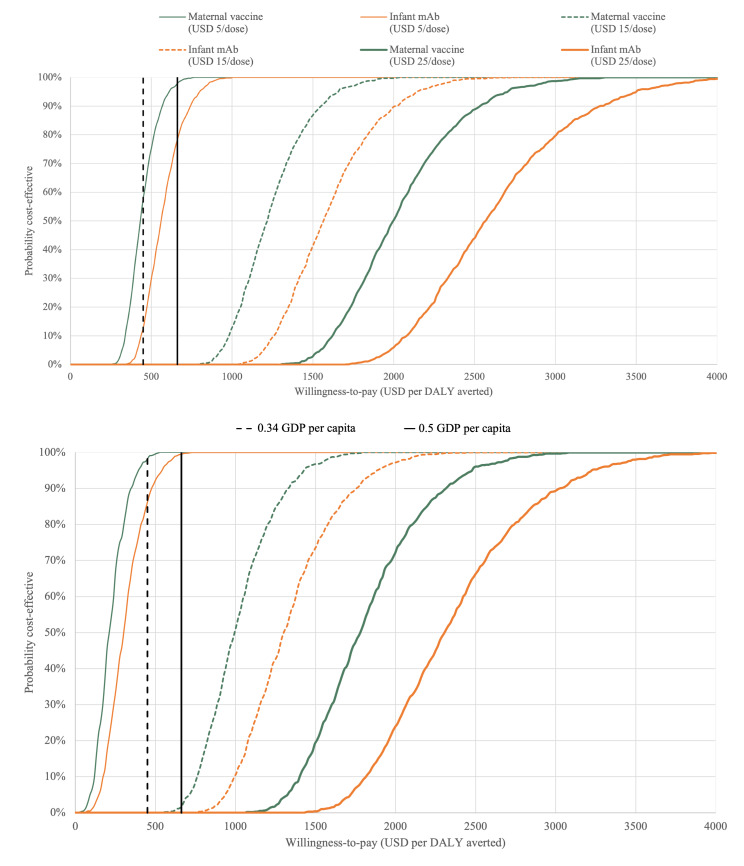
Cost-effectiveness acceptability curves for introducing a maternal vaccine or the long-acting infant mAb for RSV prevention over a range of prices per dose and cost-effectiveness thresholds. **Panel A.** Governmental health perspective. **Panel B.** Societal health perspective. DALY – disability-adjusted life year, mAb – monoclonal antibody, RSV – respiratory syncytial virus. The curves are the result of 1000 Monte Carlo simulations given base-case assumptions for the maternal vaccine and the long-acting infant mAb. The dashed black line indicates 0.34 times the gross domestic product per capita, while the solid black line shows 0.5 times the gross domestic product per capita.

At a WTP threshold of 0.34 times GDP per capita, the most cost-effective intervention strategy would need to be priced ≤USD 5 per dose to achieve >55% probability of being considered cost-effective (**Figure 3**, Panels A and B).

## DISCUSSION

Our study shows that either the maternal vaccine or long-acting infant mAb have the potential to substantially reduce the total number of RSV cases, clinic visits, hospital admissions, and deaths in Nepal. Our estimates suggest that year-round RSV passive immunisation strategies would need to be priced *<*USD 5 per dose to be considered cost-effective at the most conservative WTP threshold of 0.34 times the GDP per capita in Nepal.

While the maternal vaccine generated greater health benefits at a lower cost under our base-case assumptions, this result was influenced by key variables, including product efficacy, duration of protection, dose price, and disease burden. The mAb may become more cost-effective under alternative assumptions, particularly if timely administration at birth or a seasonal approach is implemented. Given the uncertainties around real-world implementation, both strategies should be considered if they are affordable and priced *<*USD 5 per dose. Programmatic priorities, including coverage, feasibility, and equity, should also inform the final product choice.

Our findings align with a cost-effectiveness analysis conducted in Vietnam [24], which also found that both the maternal vaccine and long-acting infant mAb have the potential to be cost-effective at USD 5 per dose, with a WTP threshold of 0.5 times the national GDP per capita. However, they found that the long-acting infant mAb is potentially more cost-effective in Vietnam, highlighting how local context can shift comparative advantages.

The seasonal approach shows that restricting administration to an eight-month window achieves comparable reductions in RSV-associated disease burden when compared to a year-round programme, while improving dose efficacy for both maternal vaccine and long-acting infant mAb. In Nepal, targeting birth months between February and September (or January to August for maternal vaccination) could result in significant cost savings. These results are in line with modelling analyses from Argentina and Mozambique, which found that implementing a seasonal approach preserved most of the health gains of year-round administration while reducing the number of doses needed [22,23].

To model mAb uptake, we used BCG coverage as a proxy, given that both interventions are delivered in early infancy through standardised immunisation programmes. In Nepal, children do not typically receive BCG immediately at the time of hospital discharge, as many health facilities provide routine immunisation only on selected days of the month due to programmatic constraints. While mAb delivery may require additional logistical inputs such as enhanced cold chain and staff training, Nepal has longstanding experience with vaccine introduction, and vaccine acceptance is generally strong [37]. Therefore, BCG coverage remains the most appropriate proxy in the absence of real-world implementation data from LMICs. This assumption was influential in the analysis and explains why the maternal vaccine appeared to be the more cost-effective option when the same dose price was assumed for both products. Both products, however, had similar cost-effectiveness when the mAb was assumed to be administered on time at birth. Realising the full benefit of the long-acting infant mAb, innovative strategies for achieving high and timely coverage of newborns are essential. Possible approaches include targeting mAb administration based on risk factors, seasonal administration, or using the mAb selectively among newborns who did not receive passive immunisation from a maternal vaccine.

We based our input parameters on the most recent clinical trial results available during the study period. Since then, an updated efficacy estimate from the MATISSE trial for the maternal vaccine has been published [38], reporting the following efficacy rates: 70.0% for severe medically attended RSV-ALRI (compared to 69.4% in our analysis) and 49.2% for non-severe RSV-ALRI (compared to 51.3% in our study). Considering the close alignment between the updated values and the input parameters we used, we do not anticipate any substantial impact on the model's outcomes.

Mortality estimates remain one of the more uncertain parameters in modelling, particularly in LMICs where vital registration systems are incomplete. We selected the higher mortality estimate from the bottom-up approach for the base case to better reflect the likely true burden of disease in this context. To assess the impact of this assumption, we conducted scenario analyses using both the lower and higher mortality estimates based on an even more conservative lower bound from Li *et al*. As expected, using lower mortality assumptions reduced projected health gains and led to a moderately less favourable cost per DALY averted. However, the overall conclusions regarding the relative cost-effectiveness of the two strategies remained consistent. These findings underscore the importance of strengthening mortality surveillance to better inform future evaluations.

Nepal has made substantial progress in enhancing its immunisation programme by integrating the typhoid conjugate vaccine into its routine vaccination schedule. Following a major earthquake in November 2023, targeted vaccination campaigns reached over 118 000 children with the Measles-Rubella vaccine and 520 000 individuals with the typhoid conjugate vaccine. The country’s vaccine delivery infrastructure, built on strong leadership and governance, healthcare finance, and human resources, provides a solid foundation for introducing RSV immunisation strategies. Integrating maternal RSV vaccination with existing maternal immunisation (*e.g.* tetanus, diphtheria, and pertussis) may further enhance coverage and increase community acceptance. Moreover, Nepal’s high routine immunisation coverage and previous success in introducing vaccines against *Haemophilus influenzae* type b, pneumococcal, and, most recently, cervical cancer, demonstrate the system’s capacity and public willingness to accept new vaccines.

We assumed moderate coverage estimates for the maternal vaccine. We believe, however, that coverage rates are likely to improve significantly with comprehensive education and outreach about the vaccine's potential benefits, specifically its role in preventing early infant pneumonia. As awareness grows, these conservative assumptions may no longer hold true. In Nepal, where health expenditures are primarily paid out-of-pocket and insurance coverage remains low, RSV immunisation strategies must consider household financial burden. Preventive strategies, such as immunisation programmes fully financed by the government (which allocates about 5.8% of its annual budget to health sector spending [39]) and supported by Gavi and other international organisations, can not only reduce disease incidence, but also prevent household impoverishment as demonstrated in our analysis from a societal health perspective.

Although both immunisation strategies have so far been primarily introduced in high- and middle-income countries, new WHO guidance and Gavi support aim to support their rollout in LMICs like Nepal. Following WHO prequalification in March 2025, the maternal vaccine became eligible for Gavi support in July 2025 [13,14]. While Gavi support remains critical for vaccine introduction, it is temporary. The Government of Nepal has consistently increased its share of immunisation programme costs, and with the planned graduation to middle-income status by 2030, Gavi support will change. To prepare for this transition, the government has outlined strategies for diversifying resources in its National Health Financing Strategy 2023–33 [40]. Sustainability strategies may involve integrating RSV immunisation into the national health insurance scheme, increasing domestic funding of immunisation and exploring co-financing opportunities with other donors. Our study represents the first comprehensive evaluation of the health impact and cost-effectiveness of RSV intervention strategies in Nepal. We employed a diverse range of country-specific data with international estimates for LMICs to estimate the burden of RSV. Additionally, we incorporated local COI data from both governmental and societal health perspectives, reflecting the multifaceted economic consequences of RSV in the Nepalese context. Furthermore, we used the UNIVAC decision-support model, a user-friendly tool that allows local stakeholders to update the evaluation as more data become available.

We used a static cohort model that does not account for transmission dynamics, which is appropriate given that both RSV immunisation strategies evaluated provide passive immunisation to infants, and there is currently limited evidence suggesting that either of the interventions would interrupt transmission patterns. However, as the RSV vaccine landscape evolves, this modelling approach may need to be revisited. A dynamic model would require high-quality RSV transmission data from Nepal, particularly in a future context where additional vaccines, such as those targeting toddlers or older children become available, as these are more likely to influence RSV transmission dynamics.

In addition to the direct health and economic benefits we captured in the cost-effectiveness analysis, RSV immunisation strategies may provide important broader health system advantages. By reducing RSV-related respiratory illness, these interventions could help decrease inappropriate antibiotic use, which is a recognised contributor to antimicrobial resistance. Furthermore, reducing the RSV burden during peak seasons may ease pressure on paediatric wards, improving healthcare capacity and resource allocation.

Additionally, it is important to note that in two upper-middle-income country sites (Argentina and South Africa) of the phase III trial, a numerical non-significant increase in preterm births was observed in women who received the RSV maternal vaccine. Although the difference was not statistically significant, this safety signal led regulatory agencies to restrict vaccine administration to ≥32 weeks of gestational age. For countries like Nepal, where preterm birth rates are already high, these findings underscore the need for cautious policy consideration. Importantly, an ongoing randomised controlled trial (NCT06955728) is currently investigating the effectiveness and safety of the maternal vaccine in several African countries, providing additional valuable evidence on vaccine safety [3]. Findings from this trial will be critical in informing future regulatory and programmatic decisions.

Recent research has also identified RSV as a contributing factor to long-term respiratory conditions, including asthma and allergic disorders [41]. Furthermore, toddlers can play a significant role in spreading RSV to older adults, particularly those with Chronic Obstructive Pulmonary Disease, where RSV can lead to severe exacerbations [42]. Considering these factors may further support the societal value of RSV immunisation strategies beyond just immediate outcomes on infants. While both intervention strategies have clear potential to reduce RSV disease burden and demonstrate cost-effectiveness in Nepal, the decision to implement them will depend on various factors, such as acceptability, safety, alignment with government health priorities, and budget constraints. This analysis can help inform decision-makers of the economic consequences of introducing RSV interventions, especially once intervention prices are known.

## CONCLUSIONS

Our analysis demonstrates that both the maternal vaccine and the long-acting infant mAb have the potential to substantially reduce RSV-related morbidity and mortality in Nepal. While the maternal vaccine was generally more cost-effective under the base-case assumption, this finding was highly sensitive to several key parameters, including coverage, product efficacy and timing of administration. A seasonal approach, particularly one that aligns with Nepal’s RSV epidemiology, can further improve efficiency and reduce costs.

Given the uncertainties around real-world implementation and evolving clinical data, both strategies remain viable options. The ultimate decision will require careful consideration of programmatic feasibility, equity, safety and long-term sustainability, especially as Nepal plans to transition to middle-income status by 2030 and prepares for the eventual phase-out of Gavi support. As vaccine prices and delivery strategies become clearer, this study provides a critical foundation for informing national immunisation policy.

## Additional material


Online Supplementary Document
