# Supplementary material for: Cost-effectiveness of introducing a maternal vaccine or long-acting monoclonal antibody to prevent infant respiratory syncytial virus disease in Nepal
Source: J Glob Health. 2025 Nov 14;15:04292. doi: 10.7189/jogh.15.04292 (PMC12615004; doi:10.7189/jogh.15.04292)
Supplement: Online Supplementary Document [file jogh-15-04292-s001.pdf]

Supplementary Material

Appendix A – Respiratory syncytial virus (RSV) health burden calculations

The Universal Vaccine Decision-Support Model (UNIVAC model, version 1.7) uses population estimates from the United Nations World Population Prospects to calculate the total number of life-years lived by children under five years of age [2,5]. These life-years are then multiplied by the incidence rates of RSV-related outcomes (including cases, outpatient visits, hospital admissions, and deaths) to evaluate the expected health burden of RSV in the absence of intervention strategies, reflecting the current status quo. The rates estimated for each RSV outcome were estimated by using a range of data sources, as outlined in *Supplementary Figure 1*.

In total, we estimated approximately 244259 cases of RSV-related illness, corresponding to 7313 per 100000 under five years of age for non-severe and 963 for severe cases per year, based on a cohort study by Chu et al. and international data from Li et al. [3,4,6]. Chu et al. reported that 18.5% of RSV cases in children under six months of age in rural Nepal were classified as severe or very severe [3], while Li et al. estimated that 10% of RSV cases in children under five years old were severe ("with chest wall indrawing") [6]. Since the data from Chu only included children under the age of six months, we adjusted the incidence rate to reflect the under five years by assuming 22% of the under-five children would be under six months of age, based on preliminary estimates from a systematic review of RSV age datasets in low- and middle-income countries (LMICs) [7]. We calculated the ratio of severe cases in children under six months of age relative to the entire under-five group. This ratio was then multiplied by the 18.5% observed in Chu, resulting in 11.6% for severe cases in the overall under five population.

We assumed that 26% of non-severe RSV cases would have a clinic/outpatient visit at a formal medical public health facility based on the average of estimates reported in the Demographic and Health Survey 2022 (DHS 2022) and Multiple Indicator Cluster Surveys 2019 (MICS 2019) [8,9]. The MICS estimate refers to care-seeking for acute respiratory infection (ARI), which we used as a proxy for RSV-ARI. For severe RSV cases we assumed that 75% would have access to any healthcare facility, based on the overall percentage of children with ARI, who were treated at any healthcare provider (public or private) as reported in the DHS 2022. This is a conservative estimate, as healthcare-seeking for severe RSV is likely higher than the average for all ARI cases. We assumed that each severe RSV hospital admission would be associated with three outpatient/clinic visits. This assumption was informed by insights from healthcare providers who reported that caregivers in Nepal commonly return to healthcare facilities when symptoms persist or worsen, and that follow-up care was typically provided after hospital discharge. In the final step we applied the mid-value of the estimated case fatality rate of 0.9% from Li et al. for LMICs cases [6] and the 3% mortality rate reported by the ICU Network Study Group in Nepal [2] to estimate the number of severe RSV admissions in children under five years old that died. This

mid-value of 2% gives us 418 severe RSV related deaths. We derived the number of severe cases that resulted in death in the community by performing backwards calculations. We assumed that 67.5% of RSV deaths occur in the community in LMICs. This assumption was based on the following studies: a study by Li et al.[6], which found that 44% of RSV deaths occur in the community in LMICs, and another study from rural India that reported a 10 times higher RSV mortality in the community (91%) [10]. We also used a top-down proportional mortality approach to check the plausibility of our estimates. We assumed 17733 deaths among children <5 years, of which 13% were due to ALRI and 643 of these were attributable to RSV.

In the scenario analysis, we estimated the number of RSV cases in the under-five population by adjusting the burden estimates using a lower-bound estimate of 3700 cases from Li [6] and an upper-bound estimate of 10344 cases based on Chu [3] (25% of their estimates).

Appendix B – RSV age burden distribution

Age-burden distributions for RSV were derived from a recent cost-of-illness (COI) study [1] and a related high-dependency unit and intensive care unit study in Kathmandu, Nepal [2]. The COI data and ICU data were extrapolated to represent the <5-year age group using estimates from a systematic review of RSV age distributions in LMICs [7]. These extrapolated distributions, applied separately to non-severe, severe, and life-threatening RSV cases, were consistent with community-based RSV surveillance data in rural southern Nepal [3] and Bhaktapur [4], supporting the use of the COI and ICU data for impact modelling.

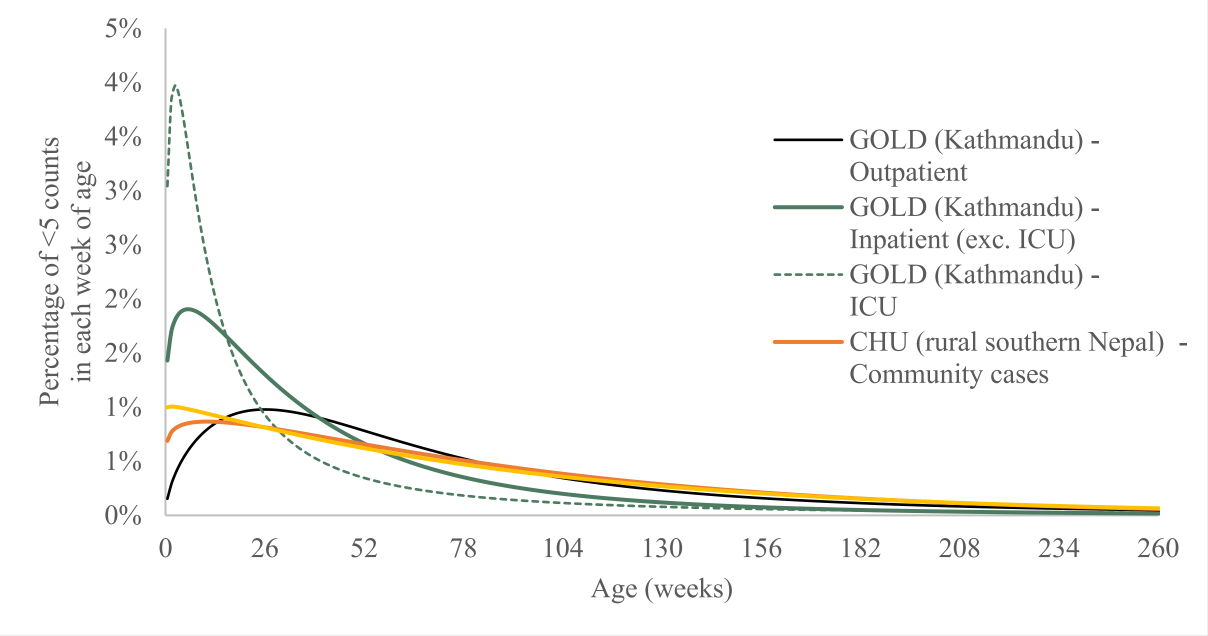

Supplementary Figure 2. Age distribution of RSV presentations in Nepal for different locations of disease presentation, from different resources. Weekly estimates were generated using fitted Burr curves to available country specific data. [1-4]
Abbreviations: ICU, intensive care unit; RSV, respiratory syncytial virus

Appendix C – RSV economic burden

RSV-related care costs were estimated for each outpatient visit (non-severe and severe) and hospital admission (severe) at public health facilities. In the base-case scenario we used cost data from a recent COI study on RSV conducted in Kathmandu, Nepal, from July 2023 to November 2023 [1]. Governmental costs included all expenses incurred by the government in providing services and infrastructure for children with an RSV infection during the full RSV illness episode, as well as direct medical costs borne by the government. From a societal health perspective, the costs included all government health costs as well as direct medical expenses (e.g., medication, imaging), direct non-medical expenses (e.g., transportation, food), and indirect costs (e.g., lost income) that were borne by households during the initial visit at a governmental health facility and prior/post visit at any healthcare facility (public or private). From the governmental health perspective, we assumed that the cost of an outpatient clinic visit and hospital admission was USD 15.78 (interquartile range [IQR]: USD15.04–USD 16.52) and USD 58.05 (IQR: USD 53.86–USD 80.87) respectively. From the societal perspective, the equivalent costs were USD 43.43 (IQR: USD 24.77–USD 62.09) and USD333.33 (IQR: USD 306.60–USD 360.60). To enhance the generalisability of the data, we weighted these estimates based on the distribution of public primary (N=340), secondary (N=17), and tertiary healthcare facilities (N=27) in the country. The relative costs of care at each level of facility were obtained from the World Health Organization Choosing Interventions that are Cost-Effective estimates for inpatient and outpatient care [11].

Appendix D – Seasonal Approach

In this supplementary analysis, we evaluated the potential health and economic impact of implementing a seasonal administration strategy for both the maternal vaccine and the long-acting infant mAb targeting infants in Nepal.

Methods

In our main analysis we conducted a series of deterministic scenario analyses, including a seasonal dose administration for the maternal vaccine and mAb. For this evaluation we used data from the RSV GOLD III – Health Economics Study in Kathmandu, Nepal, in which all hospitalised children under two years of age with a (severe) ARI diagnoses were tested for RSV during one local respiratory season (from July till November 2023) [1]. In total, 532 RSV positive hospital admissions have been recorded in this cohort. Expected protection by an immunisation strategy was estimated based on the child's date of birth and date of admission. To complement this data and to account for potential seasonal and geographic variation, we included data from a year-round prospective surveillance study conducted in Bhaktapur, Nepal, between July 2004 and June 2007 [4]. This study identified 334 RSV-positive cases of community-acquired pneumonia in children under three years of age. For comparability with the RSV GOLD study data and consistency in the targeted population, we restricted the seasonal analysis to children under two years of age. As no children under two months of age were reported in the Bhaktapur dataset, we applied the age distribution observed in the GOLD study to impute case numbers for the youngest age group and to estimate RSV burden during these months. We simulated multiple birth month cohorts to identify optimal timing for seasonal immunisation using either a maternal vaccine or a mAb. For each scenario, we estimated the anticipated reduction in RSV-associated hospital admissions and calculated dose efficiency, defined as the number of doses required to avert 1% of RSV hospitalisations. These seasonal strategies were compared to a year-round preventive strategy. To ensure comparability of health impact, outcomes for the under-two population were extracted from the UNIVAC model.

Results

Given the seasonal burden of RSV disease in Nepal, which is primarily concentrated between July and November [1,3,12], we evaluated various seasonal strategies in comparison to year-round immunisation. Our analysis in children under two years showed that an 8-month seasonal strategy targeting infants born between February and September demonstrated the best dose efficiency to avert RSV related hospitalisation by 23–32% for both maternal vaccination and mAb, depending on the assumptions for vaccine efficacy. Assuming fixed vaccine efficacy resulted in higher dose efficiency, with only 2-3% reduction in health impact compared to a year-round strategy. Overall, the improvements were similar across both interventions, though the mAb showed slightly greater benefit when gradual waning of effectiveness was assumed (24% based on STRAND; 31% based on GOLD vs 20% based on STRAND; 26% based on GOLD). (*Supplementary Figure 3*, *Supplementary Table 1 & Supplementary Table 2*)

To ensure comparability with the data inputs used, the UNIVAC model reports outcomes for children under five years. Here we also present results for the under-2 population (*Supplementary Table 3)*. Using the seasonal approach, the estimated impact for the maternal vaccine and the long-acting infant mAb in children under two was 39% and 37% for deaths and 28% and 30% for hospitalisations, respectively. Based on UNIVAC outputs, the corresponding impact in children under five years was slightly lower at 36% and 34% for deaths and 25% and 27% for hospitalisations, indicating only modest differences between the two age groups.

Discussion

Our analysis shows that a 8-month seasonal administration gives similar overall impact but improves dose efficiency compared to a year-round strategy for both the maternal vaccine and the mAb. Targeting infants born between February and September (i.e. January and August for maternal vaccine administration) could potentially result in cost savings. We observed slightly greater health benefits in the GOLD data set compared to the STRAND data set. This divergence likely reflects underlying differences in case severity, as the GOLD data are based on hospitalised RSV cases with (severe) ARI, whereas the STRAND study represents community-based RSV.

In Nepal, where seasonal administration of an immunisation is currently not implemented, introducing a seasonal approach may optimise resource allocation. However, implementation of a seasonal administration presents several challenges, including training of healthcare personnel and investment in education, logistics and monitoring systems.

For maternal vaccination, integration could be integrated in existing antenatal care (ANC) programs to facilitate implementation. ANC in Nepal is relatively well established, with many pregnant women attending one or more visits during pregnancy. Routine vaccinations, such as tetanus toxoid are already administered during these visits, providing a feasible platform to introduce maternal RSV vaccination. This integration could improve vaccine acceptance and increase coverage.

In contrast, administration of a mAb may be more challenging and potentially more expensive, particularly under a seasonal dosing strategy. Seasonal administration of a mAb requires precise planning, training and robust logistics to align with local RSV circulation patterns. These additional requirements could increase delivery costs per dose, potentially making seasonal mAb programmes more expensive overall compared to continuous year-round administration.

It is important to consider that RSV seasonality in Nepal is closely linked to the monsoon season (June–September). The majority of RSV cases occurring within this period affect infants born between February and September, meaning that a seasonal immunisation strategy should aim to protect infants both prior to and during the RSV season. While RSV transmission also occurs outside this period, infants born from October to January may have a lower risk due to residual maternal antibody protection and reduced exposure. Our analysis currently does not include catch-up doses for mAb administration.

Allowing catch-up vaccination of older children entering their first RSV season could further optimise protection and should be considered in future analyses and implementation planning. Furthermore, our seasonal analysis focused on severe outcomes and did not account for other RSV presentations (e.g. community cases, outpatient visits), which typically affect slightly older age groups and carry a separate economic burden.

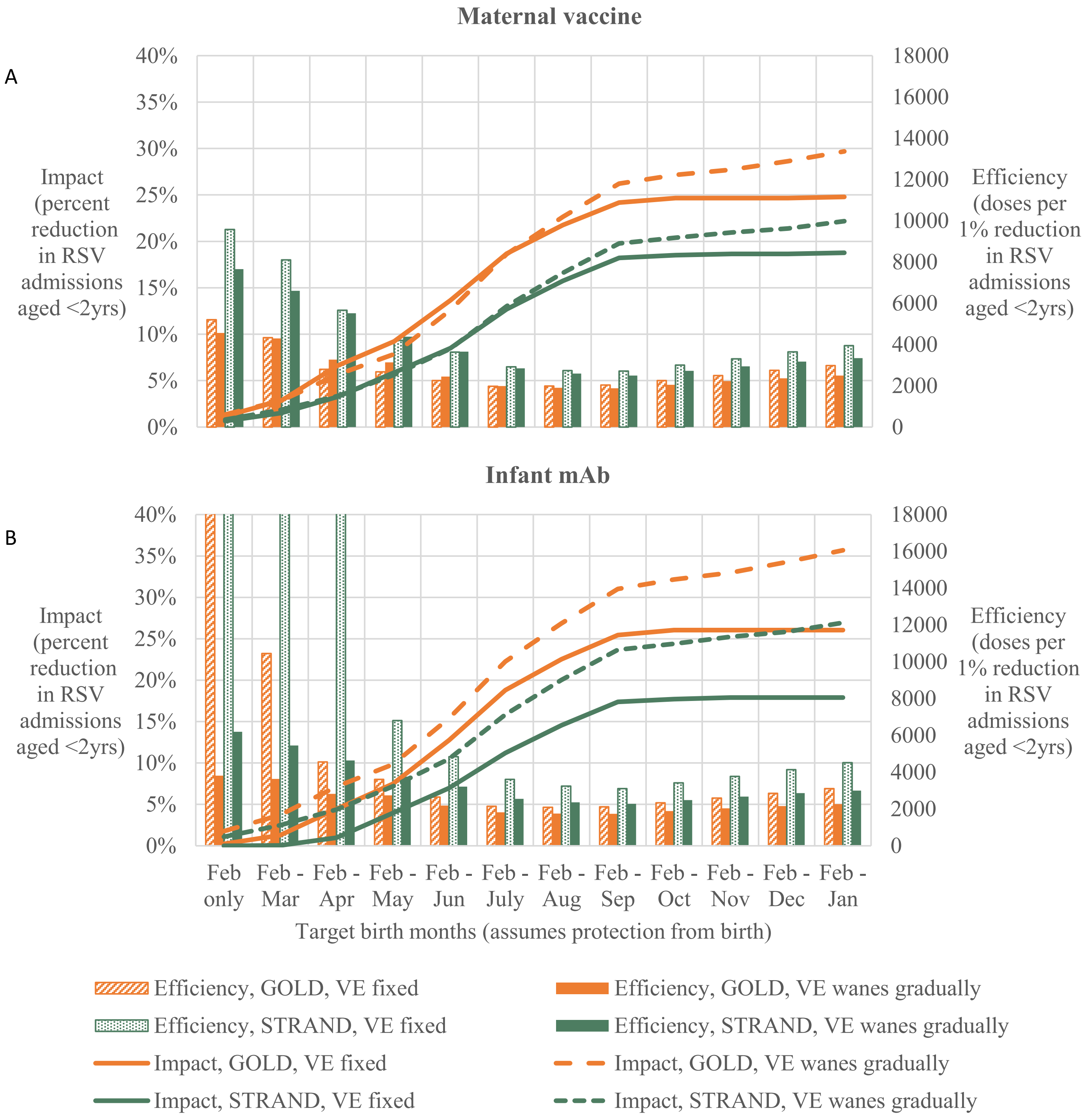

Supplementary Figure 3. Impact and efficiency of seasonal administration of maternal vaccine (A) and long-acting infant mAb (B) in children under two years of age for targeted birth months, based on data from [1] & [4].
Increasing the number of targeted birth months leads to higher impact and improved efficiency under both, fixed and waning efficacy assumptions up to an 8-months strategy (Feb-Sep). Beyond this point, impact increases only slightly, while the number of doses needed per 1% reduction in RSV admissions begins to rise again.
* For readability and consistency with panel A, the axis range has been restricted to 40%

Supplementary Table 1. Health impact and dose requirements of seasonal RSV immunisation strategies for maternal vaccine (MV) and long-acting infant monoclonal antibody (mAb) in children under 2 years old in Nepal, under fixed waning assumptions (STRAND and GOLD data sets)

Fixed efficacy												
	STRAND						GOLD					
Strategies	Reduction in admissions in under 2 years old		Doses required per 1% reduction		Change in dose efficiency compared to annual strategy		Reduction in admissions in under 2 years old		Doses required per 1% reduction		Change in dose efficiency compared to annual strategy	
	MV	mAb	MV	mAb	MV	mAb	MV	mAb	MV	mAb	MV	mAb
Feb only	1%	0%	9575	500000	< –100%	< –100%	1%	0%	5208	28863	-74%	< –100%
Feb–Mar	2%	0%	8103	279161	< –100%	< –100%	3%	1%	4327	10452	-45%	< –100%
Feb–Apr	3%	1%	5662	20762	-44%	< –100%	7%	4%	2797	4552	6%	-47%
Feb–May	6%	4%	4196	6800	-6%	-51%	9%	7%	2675	3606	10%	-16%
Feb–Jun	8%	7%	3632	4845	8%	-7%	14%	13%	2257	2647	24%	15%
Feb–Jul	13%	11%	2914	3608	26%	20%	19%	19%	1981	2151	34%	31%
Feb–Aug	16%	15%	2742	3241	31%	28%	22%	23%	1983	2093	34%	33%
**Feb–Sep**	**18%**	**17%**	**2709**	**3104**	**31%**	**31%**	**24%**	**25%**	**2040**	**2118**	**32%**	**32%**
Feb–Oct	19%	18%	3000	3422	24%	24%	25%	26%	2250	2329	25%	25%
Feb–Nov	19%	18%	3307	3765	16%	17%	25%	26%	2500	2588	16%	17%
Feb–Dec	19%	18%	3638	4142	8%	8%	25%	26%	2750	2847	8%	8%
Feb–Jan	19%	18%	3942	4519	-	-	25%	26%	2985	3106	-	-

Supplementary Table 2. Health impact and dose requirements of seasonal RSV immunisation strategies for maternal vaccine (MV) and long-acting infant monoclonal antibody (mAb) in children under 2 years old in Nepal, under gradually waning efficacy assumptions (STRAND and GOLD data sets)

Waning efficacy												
	**STRAND**						**GOLD**					
**Strategies**	**Reduction in admissions in under 2 years old**		**Doses required per 1% reduction**		**Change in dose efficiency compared to annual strategy**		**Reduction in admissions in under 2 years old**		**Doses required per 1% reduction**		**Change in dose efficiency compared to annual strategy**	
	**MV**	**mAb**	**MV**	**mAb**	**MV**	**mAb**	**MV**	**mAb**	**MV**	**mAb**	**MV**	**mAb**
Feb only	1%	1%	7653	6196	< –100%	< –100%	1%	2%	4564	3812	-83%	-68%
Feb–Mar	2%	2%	6607	5451	-98%	-82%	3%	4%	4293	3636	-72%	-60%
Feb–Apr	3%	4%	5518	4633	-65%	-54%	6%	7%	3266	2824	-31%	-25%
Feb–May	6%	7%	4380	3759	-31%	-25%	8%	10%	3136	2744	-26%	-21%
Feb–Jun	8%	10%	3654	3213	-10%	-7%	13%	15%	2444	2191	2%	3%
Feb–Jul	13%	16%	2843	2550	15%	15%	19%	22%	1988	1817	20%	20%
Feb–Aug	17%	20%	2598	2358	22%	21%	23%	27%	1907	1757	24%	23%
**Feb–Sep**	**20%**	**24%**	**2493**	**2279**	**25%**	**24%**	**26%**	**31%**	**1882**	**1739**	**25%**	**23%**
Feb–Oct	20%	24%	2723	2486	18%	17%	27%	32%	2044	1886	18%	17%
Feb–Nov	21%	25%	2942	2673	12%	11%	28%	33%	2225	2045	11%	10%
Feb–Dec	21%	26%	3172	2869	5%	5%	29%	34%	2368	2165	5%	5%
Feb–Jan	22%	27%	3337	3003	-	-	30%	36%	2492	2267	-	-

Supplementary Table 3. Estimated health impact of RSV preventive interventions in Nepal (2025–34) for one birth cohort compared with no pharmaceutical intervention. Results are shown for children under five years and under two years old.
Assumptions: maternal vaccine (USD 5/dose 81% coverage, 69.4% efficacy, 6 months protection) and infant mAb (USD 5/dose, 97% coverage, 76.8% efficacy, 5 months protection).

Outcomes	No intervention <5 years	Maternal vaccine <5 years (reduction)	mAb <5 years (reduction)	No intervention <2 years	Maternal vaccine <2 years (reduction)	mAb <2 years (reduction)
**Lifetime costs and effects**						
Non-severe RSV cases	214,831	195,835 (9%)	183,320 (15%)	164,690	145,694 (12%)	133,195 (19%)
Non-severe RSV clinic visits	55,845	50,907 (9%)	47,654 (15%)	42,811	37,873 (12%)	34,624 (19%)
Severe RSV cases	28,290	21,264 (25%)	20,723 (27%)	25,274	18,248 (28%)	17,708 (30%)
Severe RSV clinic visits	63,659	47,849 (25%)	46,632 (27%)	56,873	41,063 (28%)	39,848 (30%)
Severe RSV hospital admissions	21,210	15,942 (25%)	15,537 (27%)	18,949	13,681 (28%)	13,276 (30%)
Severe RSV deaths	902	579 (36%)	597 (34%)	822	499 (39%)	516 (37%)

RSV GOLD III – Health Economics Study Group members*

Cameroon:
Frédéric Debellut – Center for Vaccine Innovation and Access, PATH, Geneva, Switzerland
Norbert Fuhngwa – Triangle Research Foundation, Douala, Cameroon
Henshaw Mandi – Triangle Research Foundation, Douala, Cameroon

Ghana:
Rosemary Akuaku – Department of Child Health, Korle Bu Teaching Hospital, Accra, Ghana
Joycelyn Dame – University of Ghana Medical School Korle Bu Teaching Hospital, Accra, Ghana
Amma Ekem – Department of Child Health, Korle Bu Teaching Hospital, Accra, Ghana
Bamenla Goka – University of Ghana Medical School Korle Bu Teaching Hospital, Accra, Ghana
Ebenezer Ntow – Department of Child Health, Korle Bu Teaching Hospital, Accra, Ghana
Kwabena A. Osman – University of Ghana Medical School Korle Bu Teaching Hospital, Accra, Ghana

Mozambique:
Assucênio Chissaque – Instituto Nacional de Saúde, Marracuene district, Maputo, Mozambique; Instituto de Higiene e Medicina Tropical, Universidade Nova de Lisboa, Lisbon, Portugal
Nilsa de Deus – Instituto Nacional de Saúde, Marracuene district, Maputo, Mozambique
Esperança Lourenço Guimarães – Instituto Nacional de Saúde, Marracuene district, Maputo, Mozambique; Instituto de Higiene e Medicina Tropical, Universidade Nova de Lisboa, Lisbon, Portugal
Braiton Maculuve – Ministério da Saúde, Maputo, Mozambique
Elias Manjate – Faculty of Medicine, University Eduardo Mondlane, Maputo
Yara Manjate – Faculty of Medicine, University Eduardo Mondlane, Maputo
Izilda Matimbe – Faculty of Medicine, University Eduardo Mondlane, Maputo
Tufária Mussá – Faculty of Medicine, University Eduardo Mondlane, Maputo
Mirela Pale – Instituto Nacional de Saúde, Marracuene district, Maputo, Mozambique
Cesar Palha – Faculty of Medicine, University Eduardo Mondlane, Maputo
Cristina Sinussene – Faculty of Medicine, University Eduardo Mondlane, Maputo
Farida Zavala – Faculty of Medicine, University Eduardo Mondlane, Maputo

Nepal:
Ram H. Chapagain – Kanti Children's Hospital, Kathmandu, Nepal; Nepal Paediatrics Society, Kathmandu, Nepal
Rita Dhital - Nepal Paediatrics Society, Kathmandu, Nepal
Upendra Dhungana - Public Health Administrator; Ministry of Health and Population. Nepal
Prakash Joshi – Kanti Children's Hospital, Kathmandu, Nepal; Nepal Paediatrics Society, Kathmandu, Nepal
Ranju Karki - Nepal Paediatrics Society, Kathmandu, Nepal
Adita Nepali - Nepal Paediatrics Society, Kathmandu, Nepal
Uttam Paudel - Post Doctorate Researcher (Health Economics), Chulalongkorn University
Arun K. Sharma – Tribhuvan University Teaching Hospital, Institute of Medicine, Kathmandu, Nepal; Nepal Paediatrics Society, Kathmandu, Nepal
Rupesh Shrestha – Tribhuvan University Teaching Hospital, Institute of Medicine, Kathmandu, Nepal
Nirasta Thakili - Nepal Paediatrics Society, Kathmandu, Nepal

Nigeria:
Fadlulai Abdu-Raheem – Department of Paediatrics, Ahmadu Bello University Teaching Hospital, Zaria, Nigeria

Anas Abubakar – Department of Paediatrics, Ahmadu Bello University Teaching Hospital, Zaria, Nigeria
Abdullahi Aminu – Department of Paediatrics, Ahmadu Bello University Teaching Hospital, Zaria, Nigeria
Maria Ahuoiza Garba – Department of Paediatrics, Ahmadu Bello University Teaching Hospital, Zaria, Nigeria
Fatima Jummai Giwa – Department of Medical Microbiology, Ahmadu Bello University Teaching Hospital, Zaria, Nigeria
Habiba Lawal – Institute of Child Health, Ahmadu Bello University, Banzazzau, Zaria
Bernsah Damian Lawong – Department of Economics, Ahmadu Bello University, Zaria, Nigeria
Abdullahi Musa – Department of Paediatrics, Ahmadu Bello University Teaching Hospital, Zaria, Nigeria
Teddy Naddumba – Center for Vaccine Innovation and Access, PATH, Kampala, Uganda
Aira Abiola Olorukooba – Department of Paediatrics, Ahmadu Bello University Teaching Hospital, Zaria, Nigeria

Support:
Andrew Clark – Department of Health Services Research and Policy, London School of Hygiene & Tropical Medicine, London, UK
An Nguyen – Center for Vaccine Innovation and Access, PATH, Ho Chi Minh city, Vietnam
Clint Pecenka – Center for Vaccine Innovation and Access, PATH, Seattle, WA, USA

The Netherlands:
Louis Bont – University Medical Centre Utrecht, Utrecht, The Netherlands
Neele Rave – University Medical Centre Utrecht, Utrecht, The Netherlands
Farina Leonie Shaaban – University Medical Centre Utrecht, Utrecht, The Netherlands

* The authors are listed in alphabetical order of their surnames, according to the specific country teams with which they were involved in the study. Team members from University Medical Centre Utrecht, along with supporting staff, were involved at all study sites. A detailed overview of authorship contributions for each country can be found in the respective paper.

References

1 Rave N, Sharma AK, Chapagain RH, Shrestha R, Nguyen A, Pecenka C, et al. Assessing the cost of illness of RSV and non-RSV acute respiratory infections in Nepali children. 2025.

2 RSV GOLD III - ICU Network Study Group. Respiratory syncytial virus infection among children younger than 2 years admitted to a paediatric intensive care unit with extended severe acute respiratory infection in ten Gavi-eligible countries: the RSV GOLD-ICU Network study. Lancet Glob Health. 2024.

3 Chu HY, Katz J, Tielsch J, Khatry SK, Shrestha L, LeClerq SC, et al. Respiratory syncytial virus infection in infants in rural Nepal. J Infect. 2016;73:145-54.

4 Mathisen M, Strand TA, Sharma BN, Chandyo RK, Valentiner-Branth P, Basnet S, et al. RNA viruses in community-acquired childhood pneumonia in semi-urban Nepal; a cross-sectional study. BMC Med. 2009;7:35.

5 United Nations. World Population Prospects 2022: Summary of Results. 2022.

6 Li Y, Wang X, Blau DM, Caballero MT, Feikin DR, Gill CJ, et al. Global, regional, and national disease burden estimates of acute lower respiratory infections due to respiratory syncytial virus in children younger than 5 years in 2019: a systematic analysis. Lancet. 2022;399:2047-64.

7 RSV Age Study Collaborators. Age distribution of respiratory syncytial virus disease in children <5 years of age in low-income and middle-income countries: a systematic review and meta-analysis.

8 Ministry of Health and Population. Nepal Demographic and Health Survey 2022. Kathmandu, Nepal: Ministry of Health and Population [Nepal], 2023.

9 Central Bureau of Statistics (CBS). Multiple Indicator Cluster Survey 2019. 2019.

10 Simões EAF, Dani V, Potdar V, Crow R, Satav S, Chadha MS, et al. Mortality From Respiratory Syncytial Virus in Children Under 2 Years of Age: A Prospective Community Cohort Study in Rural Maharashtra, India. Clin Infect Dis. 2021;73:S193-s202.

11 Organization WH. WHO-CHOICE estimates of cost for inpatient and outpatient health service delivery. 2021.

12 Mathisen M, Basnet S, Sharma A, Shrestha PS, Sharma BN, Valentiner-Branth P, et al. RNA viruses in young Nepalese children hospitalized with severe pneumonia. Pediatr Infect Dis J. 2011;30:1032-6.
